# The Benefits of Living Without Meaning *Sub Specie Aeternitatis*

**DOI:** 10.1007/s10790-021-09839-5

**Published:** 2021-08-05

**Authors:** Peter Kügler

**Affiliations:** Department of Philosophy, University of Innsbruck, Innrain 52d, 6020 Innsbruck, Austria

## Sub Specie Aeternitatis

The phrase “sub specie aeternitatis” has been coined by Spinoza, but the idea can be traced back to Plato, who writes in the *Republic* that “the contemplation of all time and all being” (*kai theōria pantos men chronou pasēs de ousias*) can help philosophers to lose their fear of death (Plato 1991, p. 165; 486a). Our present understanding of this perspective—which I will henceforth denote as SSA—has been largely shaped by Thomas Nagel’s discussion of a topic closely related to the problem of death: the meaning of life. Nagel characterizes SSA as a standpoint that allows us to see ourselves “from outside”, eventually gaining enough distance to attain “an external view of the universe, which abstracts from our own position in it” (Nagel 1986, p. 214). This is a metaphorical description, to be sure, since we cannot literally imagine the whole universe without shrinking it to human measures and ignoring too many of its details. Thus, it may be better to consider SSA as a product of *thinking* based on physical science.

Cosmology tells us that the observable universe has an age of 13.8 billion years, which is roughly 200 million times the lifespan of a person dying at 70, the approximate average life expectancy worldwide. The universe’s diameter is estimated to be 93 billion light-years, while the diameter of the earth is merely the 742 millionth part of one light-year. My life is just a miniscule episode in this vastness of time and space, having some impact here on earth, but not mattering at all to the larger universe. So, my life seems to have no meaning when I consider it *sub specie aeternitatis*.

SSA is a perspective from which human goals, values and concerns appear so small that they lose their significance. We succeed in jobs, help other people, raise children, care for the environment and pursue our dreams; we seek love, pleasure, wisdom, money or whatever else. Some of these goals are very personal, while others are imposed on us by society and shared by almost everyone. But they only come to the fore when we look at the world not *sub specie aeternitatis*, but from a human point of view: *sub specie humanitatis*.

In what follows, I will refer to meaning *sub specie aeternitatis* as *objective* meaning. Meaning of life in this sense does not depend on human judgments and valuations. It is rather based on the assumption that values are objective. Many readers will know this type of objectivity by the name “realism”. As Nagel writes, “there is a connection between objectivity and realism, though realism about values is different from realism about empirical facts. Normative realism is the view that propositions about what gives us reasons for action can be true or false independently of how things appear to us, and that we can hope to discover the truth by transcending the appearances and subjecting them to critical assessment” (1986, p. 139).

Life must be objectively valuable—“independently of how things appear to us”—in order to have meaning *sub specie aeternitatis*. Subjective meaning, by contrast, depends on whether your life *appears* to be valuable, either to you or to other people (although you probably would not be satisfied if others told you that your life is meaningful as long as you cannot agree with their judgment). Because of the role of attitudes and judgments in assessing (or creating) subjective meaning, philosophical investigation may not be the best way to deal with it. In any case, it is not the only way, as psychology has developed methods and conceptual tools that make subjective meaning accessible to empirical research (see the overview in McDonald et al. 2012).

Psychologists often avoid the term “meaning of life” and prefer to speak of meaning *in* life, which can be “empirically examined exclusively from the perspective of the individual” (Schnell 2021, p. 5).^1^ They study how people find meaning in their lives, how they evaluate sources of meaning and how they react in experiments. For example, Heintzelman et al. (2013) have investigated the effect of coherent or incoherent stimuli, such as images of nature that do or do not follow a seasonal pattern. Although coherence of this kind is an objective feature, the focus of this research is nevertheless on the subjective *experience* of meaning. The same applies to Tatjana Schnell’s *Sources of Meaning and Meaning in Life Questionnaire*, which draws on a categorization by Robert Emmons (2003) and is intended to measure “subjectively experienced meaningfulness” (Schnell 2009, p. 488).

Meaning *sub species aeternitatis*, on the other hand, is an objective affair and is traditionally addressed in philosophy. Joshua Seachris (2013) has improved our understanding of it by distinguishing four aspects or components of SSA. One of them is the *ontological* component, which he defines as “a perspective from what is ultimately real, especially vis-à-vis normativity” (2013, p. 608). The words “especially vis-à-vis normativity” indicate that a comprehensive ontological perspective must include an account of objective values, either in the affirmative or in the negative; that is, a normative dimension to objective reality is either posited or denied.^2^

Of the other three components, I will leave aside the “modal” one, which is supposed to be “a perspective from which our lives appear to be radically contingent” (2013, p. 608). Two components remain, which we can turn into one by combining time and space. Seachris describes the spatial component of SSA as “a perspective from the spatial vastness of the universe” (2013, p. 608). Replacing the word “spatial” with “temporal” provides the time component. Together, these two yield the external standpoint alluded to above. Let us call it the *spatiotemporal* perspective.

While Seachris understands the different perspectives as “components” of SSA, we can also look at them as different *interpretations* of this concept. The ontological interpretation concerns how the world really is—the “essence of things” (*rerum essentia*), as Spinoza would have put it. In fact, the ontological interpretation can be seen as a return to Spinoza’s understanding of SSA, which has been somewhat concealed by the tradition dominated by voices ranging from Plato’s to Nagel’s.

Nowhere in Spinoza’s *Ethica* is SSA presented as an external view on the universe, if we assume that the universe can be identified with Spinoza’s one and only *substance*. For Spinoza, the core of SSA consists in the knowledge of what he calls “common notions” (*notiones communes*), which are very general—perhaps *the* most general—ideas that are adequately conceived by the mind (Powell 1906, pp. 80–83; Huenemann 2008; Steinberg 2009). Spinoza did not give a list of these ideas, and commentators do not agree as to which ideas would belong on such a list. However, Spinoza argues that the substance has infinitely many “attributes”, of which we only know two: thought and extension. These certainly belong on the list. Another candidate is the concept of law of nature, since Spinoza writes in chapter 6 of the *Tractatus theologico-politicus* (2016, p. 158) that laws of nature are conceived *sub specie aeternitatis*.

The properties represented by these and other common notions are said to be “equally in the parts and in the whole” (1985, p. 474; 2p37), so that each of them provides knowledge of an essence of the entire world. This definitely is an ontological perspective in Seachris’s sense—and one that excludes objective values, since Spinoza explains at length that “good”, “evil” and other normative concepts are merely *prejudices*, and that we do not “desire anything because we judge it to be good; on the contrary, we judge something to be good because we strive for it, will it, want it, and desire it” (1985, p. 500; 3p9s).

In the next section, I will support Seachris’s claim that, contrary to prevailing opinion, the spatiotemporal perspective is not relevant for answering the question whether life has objective meaning. We must rather turn to the ontological reading of SSA to answer that question. Section 3 afterwards will pursue Spinoza’s assumption, shared by many modern thinkers, that there are no objective values. If nothing is objectively valuable, life is not objectively valuable either and thus cannot have objective meaning. I will consider four advantages of this conclusion for human life in sections 3.1 to 3.4.

## Taking the Right Perspective

Some philosophers simply *identify* SSA with its spatiotemporal interpretation. For example, parts of Seachris’s article are dedicated to a criticism of Iddo Landau, who characterizes SSA as a perspective from which “we examine our lives in context of the cosmos at large” (Landau 2011, p. 727). Landau cites other philosophers besides Nagel who have given similar definitions of SSA: Simon Blackburn, Frank Ramsey, Nicholas Rescher, Albert Camus and William Lane Craig (in the order of appearance in the text). They all agree that SSA reveals the absence of meaning, at least if religion, and supernaturalism in general, are kept out of play. Therefore, some of them either propose to retreat to an evaluation of life *sub specie humanitatis* or suggest a theistic worldview.

Landau comes to a different conclusion, though, arguing that life can appear as meaningful even *sub specie aeternitatis*, provided that we are ready to lower our “standards of meaningfulness”. If we construe meaning in terms of our effects on the world, which obviously are very limited on a cosmic scale, one way to lower the standards is to become content with this limitation. But Landau rightly notes that the standards can also be lowered by completely separating meaning from effects: “Standards of meaningfulness may have to do not only with a moderate effect, but even with no *effect* at all; they may have to do, for example, with courage in the face of pain, failure, and disappointment, or with reaching a certain degree of wisdom, or happiness, or aesthetic fulfillment in oneʼs life, even if such achievements did not affect anyone else. And all of that can also be seen from the wide, cosmic perspective” (Landau 2017, p. 96).

In section 3.1, we will return to the role of effects in providing meaning to life. At the moment, we want to know what is involved in lowering the standards of meaningfulness. It is as if, after realizing the absence of meaning from the cosmic perspective, I “zoom into” the universe until I can see the details of my life. Discovering that it is enough to have influence only on the world around me, or to develop traits such as wisdom and happiness, I give up the pretension that my life must have cosmic meaning. Does this show that the meaning of my life can also be spotted *sub specie aeternitatis*, once we have lowered the standards of meaningfulness?

The problem with Landau’s solution is that it is indistinguishable from a change of perspectives. To opt for a lower “standard” of meaningfulness is to leave SSA behind and to take the human perspective. This also becomes evident from Landau’s examples: besides mentioning Mozart, Einstein and Tolstoy as people who led meaningful lives, he writes that “many parents who, owing to the fruits of their parenting, see their children flourishing consider their lives meaningful even if they fully recognize that their efforts did not affect the cosmos at large” (2011, p. 729).^3^ It seems that these parents simply take the human point of view—as everyone would expect them to do—and find meaning in caring for their children. This is in line with Susan Wolf’s contention that meaning is based on “reasons of love”, to which we will come in section 3.2.

However, although Landau’s strategy does not keep the promise of retaining SSA, it points in the right direction, as it indicates that the spatiotemporal perspective is not the relevant factor when it comes to the meaning of life. Seachris gives a strong argument to this conclusion when he reminds us that, although a *naturalist* will judge that life has no meaning when considering the universe *sub specie aeternitatis*, life will not appear in this way to a *theist* (Seachris 2013, p. 613). When theists look at the universe “from outside”, they believe that an omniscient God cares about every part of it, even the smallest, and evaluates every human action. Moreover, by obeying God’s commandments, the believer becomes indirectly attached to the whole universe. Since the answer to the question of whether life has meaning depends on whether the person answering it is a theist or a naturalist, the spatiotemporal perspective in itself must be neutral with regard to this question. Seachris infers from this that the ontological perspective is the decisive one.

Seachris’s argument seems convincing, but Landau has replied to it in the following way: “the fact that theists who consider life to be meaningful under the SSA perspective rely on a deep ontological-normative notion about what is most fundamental and real (that is, God) does not prove that atheists who consider life as having no meaning under the SSA perspective also rely on a deep ontological-normative notion about what is most fundamental and real” (Landau 2013, p. 462). What Landau has in mind is that atheists may deny the meaning of life for various reasons—for example, because they think that their lives have no impact on the universe at large, or because “no one will remember them a million years from now” (Landau 2013, p. 462). According to Landau, these reasons need not include any *ontological* convictions.

What he overlooks, however, is that atheism already *is* an ontological perspective by Seachris’s definition, since denying God’s existence certainly does concern “what is ultimately real”. Moreover, atheists who doubt the meaning of life often rely on other ontological convictions beyond the non-existence of God. Typically, their concern is not their missing impact on the universe, but rather the absence of objective values, an ontological assumption covered in Seachris’s account by the term “normativity”. We have already met this assumption in Spinoza, who historically has often been associated with atheism. A well-known atheist who thought in a similar way was Albert Camus: “Belief in the meaning of life always implies a scale of values, a choice, our preferences. Belief in the absurd, according to our definitions, teaches the contrary” (Camus 1975, p. 59).

## The Benefits of No Objective Values

The ontological reading of SSA concerns how reality objectively is. If we cannot find values there, we infer that human life has no objective value either. This insight has evoked some rather dramatic reactions in the past. Camus linked the absence of meaning to the only “truly serious philosophical problem and that is suicide” (1975, p. 11). David Benatar, to take a more recent example, draws conclusions that are less disturbing than suicide, but would still be unfortunate for humankind: “The absence of cosmic meaning may provide one with a reason to regret oneʼs existence or to desist from perpetuating the whole pointless trajectory by abstaining from bringing new people into existence” (Benatar 2017, pp. 53–54).

Pessimistic attitudes like these can easily obscure the fact that the non-existence of objective values has its advantages as well. I will address four of them in the following. First, the absence of objective values and objective meaning provides a reason for concentrating on *subjective* meaning and for construing it in a comprehensive way, without restricting it to goals and to effects on the world. Second, to know that nothing is objectively valuable helps to defy *elitism* about human values. Third, knowing that life has no objective meaning makes *death* seem a bit less bad than it would be if it were the loss of something objectively important or valuable. Fourth, this knowledge may completely shift our attention from questions of meaning to how we lead our lives in the *present*.

### A Richer Notion of Subjective Meaning

We have seen that Landauʼs proposal to lower the standards of meaningfulness boils down to the suggestion that we focus on the human point of view. Obviously, abandoning SSA does not yet tell us how to choose among the many options that the human point of view reveals. Landau emphasizes modest types of purposes, such as raising children. Although these activities can be very challenging, as many parents will know, they do not go beyond the personal sphere. Nevertheless, raising children transcends the parentʼs *self*, which suffices to show that self-transcendence does not require objective values and is also possible *sub specie humanitatis*.

Moreover, when my goals are large enough, they will inevitably require cooperation with other people and thus a certain degree of self-transcendence on my part. Such goals need not be unreasonably large, as there is much space between personal and family life on the one side, and utopic goals on the other. One challenge located in this middle ground that presently comes to mind is the fight against climate change, to which everyone can contribute through private behaviour and political activity. The goal of preserving a liveable environment for future generations is sufficiently close to being “objective” so as to provide life with meaning of a self-transcendent kind.^4^

What is misleading about this account, however, is that it gives the impression that *only* self-transcendence can make life meaningful. This high valuation of self-transcendence is widespread in philosophy, and arguably it is rooted in the traditional preference for SSA. Philosophical accounts of meaning inherit from SSA the idea that we must create, or relate to, values that exist independently of us, which in turn has led philosophers to associate this meaning with our impact on the world and on other people.

Berit Brogaard and Barry Smith, for example, claim that a meaningful life has a certain pattern “which relates not merely to what goes on inside the person’s head, but which involves also, in serious ways, the person having an effect upon the world” (2005, p. 443). According to Ingmar Persson and Julian Savulescu, “the meaning of our lives should be understood in terms of what we intentionally bring about in the way of outcomes of (positive or negative) value for ourselves or others” (2019, p. 229). In a similar vein, Terry Eagleton speaks of “a Western ideology for which what matters is the meanings we stamp on the world and others for our own ends” (2008, p. 71). It would be premature to think that these descriptions are equivalent: the “effects” in the first quotation need not be the same as the “outcomes” in the second and the “ends” in the third. However, what all three accounts share is the conviction that a meaningful life must have some *impact* that is valued by someone.

Eagleton wants to overcome the alleged “ideology” by placing happiness and love at the centre of life (pp. 95–98). This accords well with Landau’s view cited in the previous section, that meaningfulness need not be related to effects. If we accept what Eagleton and Landau suggest, we can avoid a too narrow view of subjective meaning biased towards effects. In addition, we get closer to how people without philosophical training think about the meaning in their lives–which takes us back to psychological research.

I have already mentioned the *Sources of Meaning and Meaning in Life Questionnaire*, which clearly reflects that non-philosophers find meaning in many different purposes and activities. The items of this questionnaire are assigned to four “dimensions” of meaning, one of which is self-transcendence (subdivided into “vertical” and “horizontal”). The dimension that is probably most distant from self-transcendence through goals and effects is called *well-being and relatedness*: “Cultivating and enjoying life’s pleasures in privacy and company” (Schnell 2009, p. 488). Together, the dimensions cover twenty-six “sources of meaning”, including some that do not necessarily depend on self-transcendence, such as “fun”, “comfort”, “freedom” and “individualism”.^5^

### Avoiding Elitism About Values

Susan Wolf claims that philosophical models of human motivation have emphasized either reasons of self-interest (such as the desire to be happy) or reasons of morality (such as the wish to reduce suffering). Both kinds of reasons motivate actions that seem to give meaning to life. Similarly to Eagleton, however, Wolf argues that what makes life meaningful is a *third* category comprising “reasons of love” (Wolf 2010, p. 4–7), to which belong such motives as raising children or helping a friend. This threefold classification of reasons—self-interest, morality, love—is compatible with the denial of objective meaning, since acting on reasons of love does not depend on objective values.

Yet, Wolf goes beyond subjective meaning by advocating what she calls the “Fitting Fulfillment View”, which says that “a life is meaningful insofar as its subjective attractions are to things or goals that are objectively worthwhile” (2010, pp. 34–35). This poses the question as to which things or goals are objectively valuable. A survey of Wolf’s account shows that she treats some human activities as more worthwhile than others, for example, when she refers to “the observation that some projects, such as rolling a stone uselessly up a hill, making handwritten copies of *War and Peace*, solving Sudoku puzzles, or caring for one’s pet goldfish, were in some way inadequate” (p. 36).

Putting aside the first two cases—who would deliberately play Sisyphus or transcribe over a thousand pages?—the question remains why Sudoku puzzles and goldfishes should not make a person’s life meaningful if that person values them highly enough. Is Wolf not displaying intellectual prejudice when she writes that “a life spent caring for a pet goldfish or entering lawn mower races is not likely to be as meaningful as one spent writing symphonies or directing a youth group” (p. 119)?

Even though I raise this question here with regard to Wolf, her position is rather typical of philosophers who tend to rate the pleasures of the intellect higher than the pleasures of the senses. John Stuart Mill’s famous statement that it is “better to be Socrates dissatisfied than a fool satisfied” (1863, p. 14) comes to mind. Whatever Mill meant by a “fool”, it is condescending to dismiss the passions of Sudoku puzzlers, goldfish lovers and lawn mower racers as devoid of meaning. Wolf’s “intuitive judgments of individual lives” (2010, p. 119) betray a preference for the values of intellectual “elites”. The assumption of value objectivity finally converts this preference into a purportedly objective truth.

To be fair to Wolf, she does acknowledge that the Fitting Fulfillment View gives the impression of elitism, which would be based on her own “bourgeois American values” (p. 39). But she defends herself against this charge by stating that neither philosophers nor any other groups “have any special expertise that makes their judgment particularly reliable”, and that value questions “are open to anyone and everyone to ask and to try to answer”. Critics of value objectivity will happily agree with this qualification but will also ask what the point is of postulating the existence of objective values if we cannot identify them with some certainty. We all know that value judgments of different people can contradict each other, and very often there is no consensual strategy for determining which of these judgments is objectively true.

In addition, it is straightforward to explain why one’s own values *seem* to be the objective ones in contrast to those of other people. This is because they have become our “second nature”, to borrow the term that has been used to denote the Aristotelian “habit” (*ethos*), and which is also linked to “character” (*ēthos*, written with an *eta*). We have acquired these values in the formative years of childhood and youth, or we have invested so much time and energy in them as adults that we can no longer think of ourselves as not pursuing them. Our second nature makes us believe that our preferences belong not to us, but to the world outside. As John McDowell puts it, “To use the rhetoric of ethical realism, second nature acts in a world in which it finds more than what is open to view from the dehumanized stance that the natural sciences, rightly for their purposes, adopt” (1998, p. 192).

We can reduce the danger of elitism if we conceive our second nature as what it is: “a cultural product; this is true whether or not it is aware of itself as such” (McDowell 1998, p. 194). Since the values that constitute a person’s second nature are culturally relative, we cannot justify a particular rating of values by claiming that it reflects an objective hierarchy; nor can we say that some activities are objectively more meaningful than others. Of course, this does not entail that the musical composer should give up this profession and become a lawn mower racer instead, or that philosophers should replace their students with goldfishes. It simply means that the composer and the philosopher are well advised to accept that other kinds of values provide meaning to other kinds of lives.

It is also important to note that the non-objectivity of values does not force us to assign the *same* value to musical composition and goldfish cultivation. Depending on the kind of music and the sophistication of the aquarium, one occupation may be more difficult to perform than the other, which may serve as a good reason for rating it higher, at least in some respects, such as financial remuneration. Nevertheless, the correlation between high difficulty and high evaluation is not an objective truth but a human convention—and, by the way, one violated in many cases.

To avoid another misunderstanding, the rejection of elitism does not imply that value judgments are not possible at all, or that every human action is *morally* acceptable. What I refer to as “elitism” is the attitude of dismissing purposes and activities as being worthless with respect to *meaning*; but meaning is just one normative aspect, morality another. You can reject the elitist attitude and nonetheless ascribe positive or negative moral value to the purposes or activities in question. A person who harms other people will be acting in a morally wrong manner even if she finds a kind of meaning in it. I am aware that some readers will not grant separating meaning from morality in this way, and that they will insist that meaning *excludes* immorality. However, we do not need to settle this issue here, since the criticism of elitism holds even under the assumption that morally wrong actions cannot contribute to life’s meaning. Provided that lawn mower racing is morally unobjectionable, it would still be an expression of elitism to dismiss this sport as meaningless.^6^

What I am suggesting is a subjectivist account of meaning, which Cheshire Calhoun calls *attitudinal subjectivist*: “Attitudinal subjectivists think that what makes an activity meaningful are mere positive personal attitudes, quite apart from the agent’s evaluative reasons for having those attitudes. You simply enjoy, feel satisfied by, care about, or love what you’re doing” (2018, pp. 40–41). Some philosophers have criticized this view for being overly permissive. Referring to Wolf (2010) and Metz (2013), Calhoun summarizes this criticism as follows: “Both Wolf and Metz, along with many others, reject attitudinal subjectivist conceptions of meaning on the grounds that *mere* subjective attitude—whether that be feelings of fulfillment, caring, or commitment—cannot by itself make a life meaningful. We cannot make a life devoted to the trivial meaningful simply by caring a lot about those trivial things” (2018, p. 28, footnote).

Although I do not think that the attitudinal subjectivist view can be dismissed by simply labelling some human activities as “trivial”, I admit that the above objection has some intuitive force. However, accepting the objection need not lead us back to objective values. Instead, Calhoun suggests that we broaden our perspective by adding intersubjectivity to subjectivity. More precisely, she distinguishes “reasons-for-me” from “reasons-for-the-initiated” and “reasons-for-anyone”. Those of the first kind are merely subjective in that they “are the reasons you have as the particular person you are” (p. 35). A lawn mower racer may simply enjoy the sport or perhaps see it as an expression of personal freedom. Both would be reasons for that very person to engage in this activity.

The other two types of reasons go beyond the personal sphere in this narrow sense. Reasons-for-the-initiated would be appreciated by all those who are familiar with the activity in question. There are probably subtleties to lawn mower racing that are valued only by the racers themselves and are unknown to people (like me) who lack in-depth information. As to reasons-for-anyone, finally, Calhoun admits that “anyone” can be limited to a certain group of people. She mentions religious believers, but as far as I can see, the reasons-for-anyone could also be those shared, say, by all lovers of motorsport in general, including lawnmower racers and spectators.

For our purposes, the important point is that neither reasons-for-the-initiated nor reasons-for-anyone presuppose the objectivity of values. Indeed, Calhoun denies that meaning is determined by “agent-independent value facts”: “Meaning is supplied by the agent’s best judgment about what those facts are, along with her reasons-for-the initiated and reasons-for-me” (p. 39). This intersubjective conception of meaning might be attractive to those convinced by the argument that the attitudinal subjectivist view is too permissive. Although I do not think this argument needs to be accepted, it is good to know that there is more than one alternative to the assumption of objective values. At least we have the choice between (merely) subjective and intersubjective values.

### Death Becoming Less Bad

I mentioned at the outset that Plato prescribed SSA as a remedy for the fear of death. In the *Republic*, he has Socrates argue that a person who contemplates all time and all being would not believe that death is something terrible, since it is impossible “that human life seem anything great [*mega*]” from that perspective (Plato 1991, p. 165). Unfortunately, experience shows that philosophical arguments alone cannot do very much against the fear of death. But they can contribute to a broader therapeutic process that also includes psychological techniques and practical exercises. Hellenistic philosophy in particular conceived of the role of philosophy in this way (Hadot 1995, ch. 3), as does modern cognitive-behavioural therapy (Robertson 2010).

One function of philosophy in this joint effort is to prove that, although the death of a person may be bad for others, it is not bad for the person dying; at least not as bad as we usually think. Epicurus famously claims that our own death is “nothing to us” because we do not experience it either during or after our lifetimes. Lucretius suggests that we need not be sad about our future non-existence any more than we are about our past non-existence. Whether or not such arguments succeed in easing the fear of death, the real question is not if death is bad in itself. What we rather need to know is whether being dead is *worse* than being alive.

Yet, this question is still too general, as there are many different ways of being alive, of which some may be better than being dead, and some may be worse. Putting aside the cases in which death comes as a relief, it is safe to say that most people prefer life to death, which arguably even applies to many of those who commit suicide. This preference for life is compatible with death being not bad in itself, because of two good things one can be worse than the other. For all we know, death could be neutral, or even good, and nevertheless be worse than a good life.

A possible reason for the belief that death is worse than life—again ignoring cases where it is preferred over life—is that it is a privation of the good. This is Thomas Nagel’s view: “When a man dies we are left with his corpse, and while a corpse can suffer the kind of mishap that may occur to an article of furniture, it is not a suitable object for pity. The man, however, is. He has lost his life, and if he had not died, he would have continued to live it, and to possess whatever good there is in living” (1970, p. 78). The standard objection to this argument is that death cannot be bad for a person who does not exist anymore. If this objection is valid, Nagel’s argument does not prove what it attempts to prove, for after death there is no longer a “suitable object for pity”.

But even if death were not a misfortune, Nagel’s account would still explain some of our attitudes towards death, especially why many people are afraid of it. Samuel Scheffler replies as follows to the question whether this fear is reasonable: “For my part, I have a hard time seeing why not, assuming that they are glad to be alive and would like to go on living” (2013, p. 102). Happy and healthy people normally prefer life over death because they value the good things life has to offer and because they think that death will deprive them of these goods. This is a reason for disliking death that can hardly be shaken by philosophical arguments, including the argument that the life we will lose is not objectively valuable and does not “seem anything great”.

However, although the absence of objective values will not make us appreciate the prospect of death, it does provide at least a small consolation by reminding us that things are good only relative to our valuation. When we think that death is worse than life, this “worse” does not express an objective ranking of good and bad. Whatever reasons we may have for disliking death, we need not worsen things by adding an objective layer to this emotion. When we consider death *sub specie aeternitatis*, it presents itself as the natural end of a biological episode that has no objective value.

The merit of this assessment of death becomes apparent when we compare it with alternative accounts that construe the process of dying as a descent from the objectively valuable condition of a living person to the worthless material condition of a corpse. In these sorts of models, dying becomes an ontological collapse, as it were, an event much more dramatic than a mere transition between physical states. In the context of supernaturalism, the classic case is the belief that the soul—understood as a spiritual part of the person—deprives the body of value when leaving it behind. But even McDowellʼs philosophy of second nature, which led him to advocate a special type of *naturalism* (McDowell 2004), implies that death is a departure from the realm of value; a departure, that is, that leaves nature “disenchanted”, a term McDowell frequently uses for the “dehumanized stance” of the natural sciences to which he refers in the passage quoted in the previous section.

There is no doubt that death is a dramatic event on both the personal and social levels, but words such as “disenchantment” and “dehumanization” only make things worse. They further dramatize the event by philosophical rhetoric. From an objective point of view, by contrast, the transition from life to death is neither good nor bad, but just the way things go. There is neither despiritualization nor disenchantment. This is a less bad prospect than if death were a loss of something objectively important or valuable.

Of course, it is difficult to consider one’s own death *sub specie aeternitatis* without subjective evaluation. Even if some sages or saints may have been able to do this, for most of us ordinary human beings, SSA can at best be a mitigation, not a cure, of the fear of death. SSA makes dying easier, but this perspective is hard to maintain over a long period of time. This section began with ancient philosophy, so let me finish it with a Stoic philosopher. One of the reasons why Marcus Aurelius wrote his *Meditations* was to remind himself of keeping an objective perspective on life, “waiting for death with a confident mind, since it is nothing but the dissolution of the elements of which every living creature is composed” (2013, p. 13; 2.17).

### Living in the Present

When Ludwig Wittgenstein contemplated the meaning of life *sub specie aeternitatis* and realized that there is no such meaning, he solved the problem in a different way. He first addressed it in his notebooks of the time when he worked on the *Tractatus*. In these remarks, the problem of meaning appears in a supernaturalist setting: “To believe in God means to see that life has a meaning” (Wittgenstein 1979, p. 74; 8 July 1916). In the *Tractatus* itself, God plays a peripheral part and Wittgenstein defends naturalism, the notion of meaning thereby becoming clearly associated with SSA. Since Wittgenstein likely took the idea of SSA from Schopenhauer (1966, § 34) and was unaware of Spinoza’s ontological conception, he alludes to the spatiotemporal reading and understands SSA as a contemplation of the world “as a limited whole” (Wittgenstein 1922, p. 187; § 6.45).^7^

That Schopenhauer was the source of Wittgenstein’s understanding of SSA has profound consequences. In effect, the spatiotemporal reading is turned upside down, as SSA loses its cosmic significance and becomes a view from the perspective of the *present*. Both Schopenhauer and Wittgenstein construe this “presentist” perspective, as we may call it, as an awareness of individual things on the verge of identification. Referring to the contemplation of natural objects, Schopenhauer writes, “we are no longer able to separate the perceiver from the perception, but the two have become one, since the entire consciousness is filled and occupied by a single image of perception” (1966, pp. 178–179). In Wittgenstein’s notebooks, the object filling his consciousness is a stove: “But if I was contemplating the stove *it* was my world, and everything else colourless by contrast with it” (1979, p. 83; 8 October 1916).

Conceptually speaking, this transformation of the spatiotemporal into the presentist perspective was made possible by the fact that “eternal” can mean “temporally infinite” as well as “being outside of time”, and that *being in the present* can be seen as a way of being outside of time. Wittgenstein suggests the latter in § 6.4311 of the *Tractatus*: "If by eternity is understood not endless temporal duration but timelessness, then he lives eternally who lives in the present" (1922, p. 185). In the *Notebooks*, he has already given a hint on how to read this remark, claiming that “the man is fulfilling the purpose of existence who no longer needs to have any purpose except to live. That is to say, who is content” (1979, p. 73; 6 July 1916).^8^

Emyr Thomas understands Wittgenstein’s presentist approach to SSA as an expression of self-renunciation aiming at independence of the world (Thomas 1995, p. 330). This is certainly true insofar as the subject who focusses his or her attention on the present situation turns it away from the rest of the world, including the remote past and the remote future. In another article, Thomas (1999) reads Wittgenstein’s later philosophy—particularly in the *Philosophical Investigations*—as a *return* from renunciation to immersion. But this account of Wittgenstein’s development somewhat blurs the fact that renunciation—as immersion in the present—already is a kind of immersion in the world. Not only is the present moment a part of the world; some would even say that concentrating on the present is the deepest kind of immersion in the world that a person can possibly reach.

According to the psychologist Mihaly Csikszentmihalyi, this state of consciousness enables experiences of *flow*, which he defines as “the holistic sensation that people feel when they act with total involvement” (1985, p. 36). Csikszentmihalyi also refers to “autotelic” activities—activities that serve no purpose besides themselves. To the extent that living in the present is incompatible with thinking of the future, states of immersion, flow experiences and autotelic activities rule out such “higher” purposes as finding the meaning of life. In general, of course, it is not advisable to forget about the future, and it is hardly possible at all if you are not a hermit but earn your living in modern society. Flow and autotelic activities will always be exceptions; yet Csikszentmihalyi argues that they belong to those parts of life we enjoy most.^9^

As indicated in section 3.1 above, one way of dealing with the idea that life has no objective meaning is to exploit the wide spectrum of subjective meaning. Wittgenstein’s approach to SSA adds another aspect to this strategy: instead of worrying about the meaning of life, we can increase it by cultivating flow experiences and autotelic activities. As the famous statement in § 6.521 of the *Tractatus* says, “The solution of the problem of life is seen in the vanishing of this problem" (1922, p. 187). When you are in flow, you simply have no time to philosophize on the meaning of life—unless you are a philosopher who experiences flow when thinking about meaning.

## Conclusion

Philosophers have tended to treat SSA as an external view on the universe (section 1), but this spatiotemporal reading of SSA is not the relevant one when it comes to the meaning of life. It is therefore wise to follow the example of Spinoza and consider SSA as an ontological conception of what is ultimately real (section 2). I have investigated four benefits of the non-existence of objective values and of meaning *sub specie aeternitatis*. It paves the way for a richer notion of subjective meaning, based not only on self-transcendence and effects on the world (section 3.1); it helps to avoid elitism with regard to values (3.2); it makes death seem less bad than it would be if it were the end of something objectively valuable (3.3); and it can motivate us to live in the present moment (3.4).

This is not to say, of course, that we should always maximize subjective meaning, respect non-elitist values, contemplate death or live in the present moment. We often have good reasons, including moral ones, for not doing so. All I wanted to show is that the absence of meaning *sub specie aeternitatis* has its good sides, too, and that it is therefore less deplorable than one might think.

## Footnotes

1 In philosophy, terminology is not uniform and the distinction between “in” and “of” is frequently not made. If it is, it does not necessarily express the difference between subjective and objective meaning.

2 For this reason, Seachris writes “ontological” with the subscript “Normative”, which I have removed for brevity.

3 Landauʼs article was rewritten into chapter 7 of Landau (2017), where the example of the parents can be found on page 96.

4 At the time of writing this essay, the worldwide COVID-19 crisis offers another example.

5 What these terms mean can be derived from the associated questions. The questionnaire is available on the website of Schnell’s book (2021): https://www.routledge.com/The-Psychology-of-Meaning-in-Life/Schnell/p/book/9780367415853 (retrieved 2 February 2021).

6 Perhaps this is not the best example, because burning fossil fuel in this way could be considered morally problematic in times of climate change. But there are other seemingly “meaningless” yet morally acceptable activities that are regarded as meaningful by the people involved.

7 See also the notebook entry of 7 October 1916: “The usual way of looking at things sees objects as it were from the midst of them, the *view sub specie aeternitatis* from outside” (Wittgenstein 1979, p. 83).

8 Søren Kierkegaard may also have influenced Wittgenstein’s view of the present, especially statements from *The Concept of Anxiety* such as this one: “The present is the eternal, or rather, the eternal is the present, and the present is full” (1980, p. 86). It is likely that Wittgenstein read Kierkegaard at the time in question, although he did not mention him in writings until later (Schönbaumsfeld 2007, ch. 1).

9 Since many people regard happiness as the essence of life’s meaning, it is also worth noting that empirical evidence suggests a positive correlation between flow experiences and self-ascriptions of happiness (Csikszentmihalyi and Wong 2014).
